# Supplementary material for: Post-learning replay of hippocampal-striatal activity is biased by reward-prediction signals
Source: Nat Commun. 2025 Nov 24;16:10394. doi: 10.1038/s41467-025-65354-2 (PMC12644820; doi:10.1038/s41467-025-65354-2)
Supplement: Supplementary file 1 — Supplementary Information [file 41467_2025_65354_MOESM1_ESM.pdf]

Supplementary results

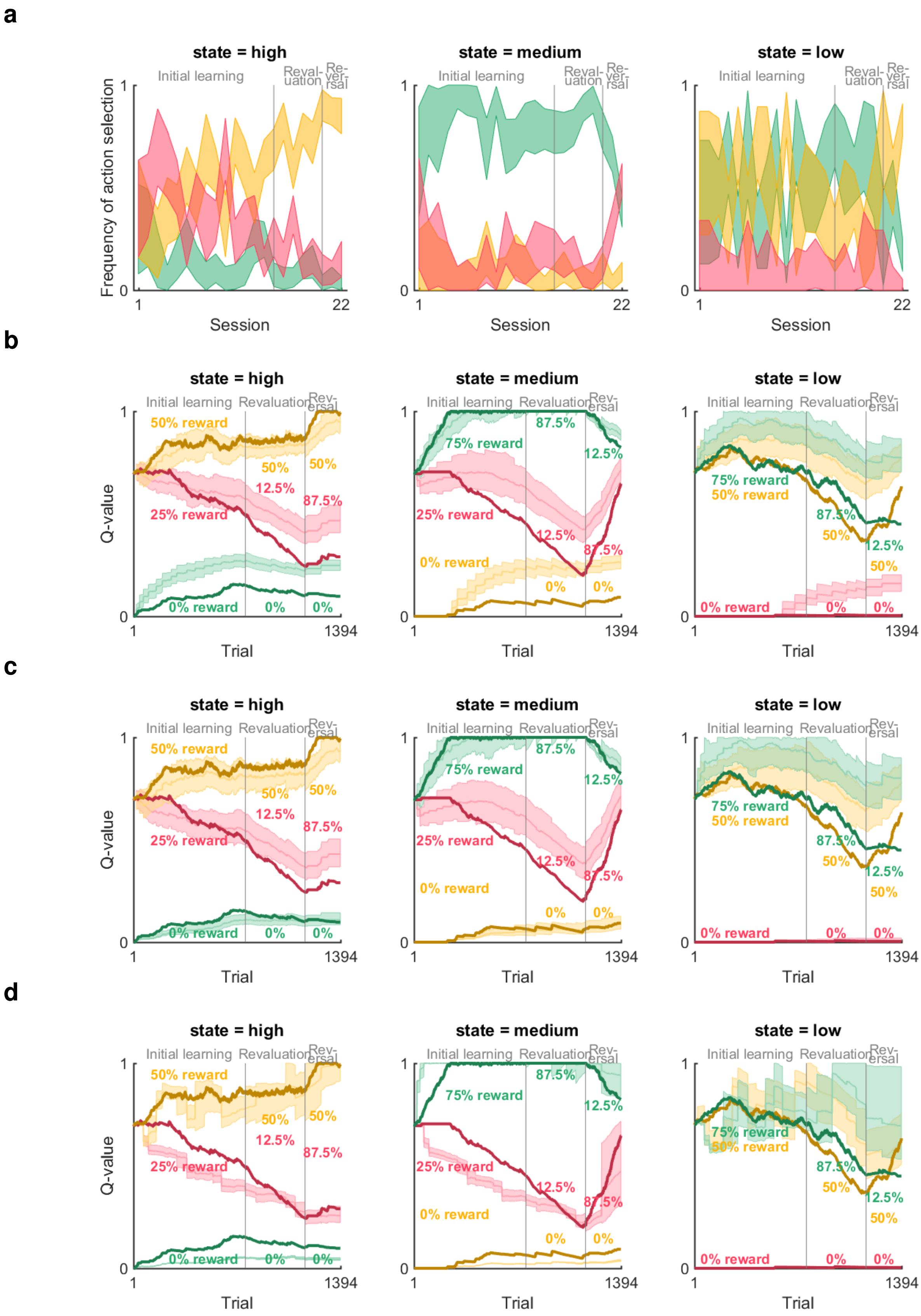

Figure S1: Example evolution of modelled Q-values across learning, for one animal and three replay policies. **a.** Frequency of actions taken in each state. Shaded regions represent 75% confidence intervals (Clopper-Pearson binomial proportion intervals). **b-d.** Range of Q-values (shaded regions) produced by 1,000 simulations, replaying 100 samples per session according to the random (b), reward-biased (c) and RPE-prioritised (d) policies, contrasted with no-replay baseline in dark lines. Colours indicate the action associated with the Q-value (high-, medium- or low-probability arm). Overlaid text indicates the changing probability of reward at each arm over the course of learning. Source data for a-d are provided as a Source Data file.

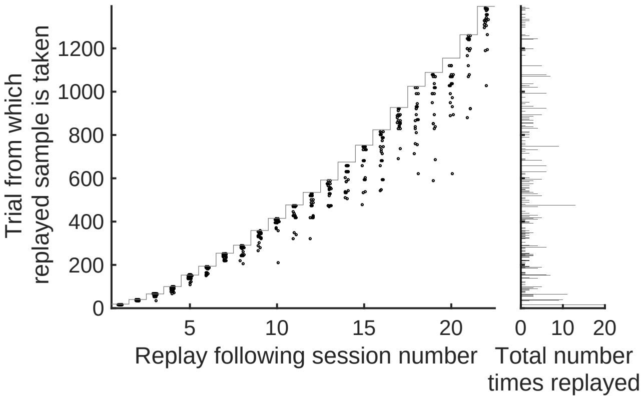

Figure S2: Trials replayed under the RPE-prioritised policy for one animal. Data points indicate trials which were replayed for a single simulation with 20 replay samples following each session. Stairs indicate the cumulative nmber of trials following each session, i.e. data points close to the stair are replays of the most recent trials. Source data are provided as a Source Data file.

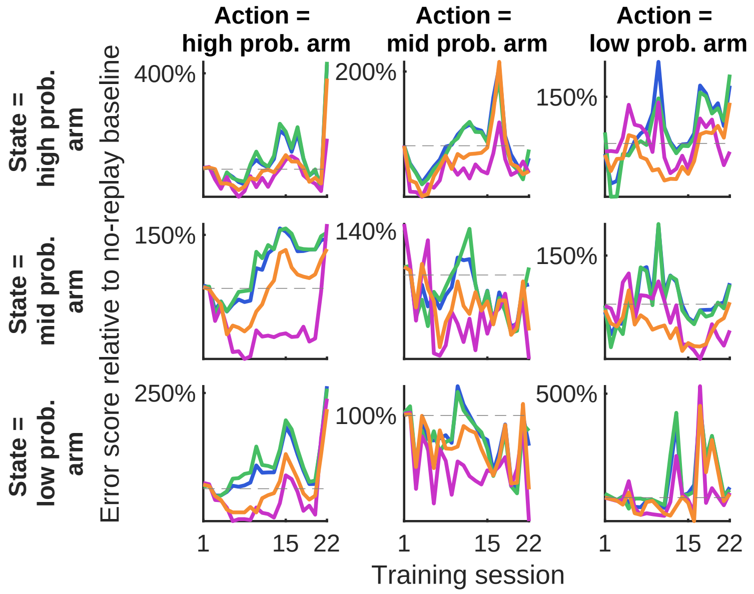

Figure S3: Change in error score for all trials on which a given state-action pair was expressed, with 15 samples replayed, relative to no-replay baseline, averaged across six animals. Intersection of "State = high prob. arm" and "Action = mid prob. arm" indicates a transition from high-probability arm to mid-probability arm. Source data are provided as a Source Data file.

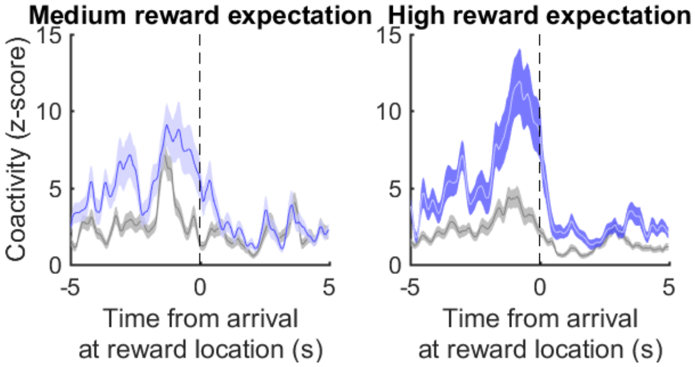

Figure S4: Mean ± s.e.m. z-scored coactivity of 129 reactivated CA1-vStr cell pairs (blue) and 293 non-reactivated CA1-vStr cell pairs (grey) around the time of arrival at reward locations on rewarded medium- and high-expected reward trials (rewarded outcomes only). Source data are provided as a Source Data file.
