# Supplementary material for: Post-learning replay of hippocampal-striatal activity is biased by reward-prediction signals
Source: Nat Commun. 2025 Nov 24;16:10394. doi: 10.1038/s41467-025-65354-2 (PMC12644820; doi:10.1038/s41467-025-65354-2)
Supplement: Supplementary file 2 — Reporting Summary [file 41467_2025_65354_MOESM2_ESM.pdf]

nature portfolio

Corresponding author(s): Emma Roscow

Last updated by author(s): Sep 24, 2025

Reporting Summary

Nature Portfolio wishes to improve the reproducibility of the work that we publish. This form provides structure for consistency and transparency in reporting. For further information on Nature Portfolio policies, see our Editorial Policies and the Editorial Policy Checklist.

Statistics

For all statistical analyses, confirm that the following items are present in the figure legend, table legend, main text, or Methods section.

n/a	Confirmed	
☐	☒	The exact sample size (*n*) for each experimental group/condition, given as a discrete number and unit of measurement
☐	☒	A statement on whether measurements were taken from distinct samples or whether the same sample was measured repeatedly
☐	☒	The statistical test(s) used AND whether they are one- or two-sided<br>*Only common tests should be described solely by name; describe more complex techniques in the Methods section.*
☐	☒	A description of all covariates tested
☐	☒	A description of any assumptions or corrections, such as tests of normality and adjustment for multiple comparisons
☐	☒	A full description of the statistical parameters including central tendency (e.g. means) or other basic estimates (e.g. regression coefficient) AND variation (e.g. standard deviation) or associated estimates of uncertainty (e.g. confidence intervals)
☐	☒	For null hypothesis testing, the test statistic (e.g. F, t, r) with confidence intervals, effect sizes, degrees of freedom and P value noted<br>*Give P values as exact values whenever suitable.*
☐	☒	For Bayesian analysis, information on the choice of priors and Markov chain Monte Carlo settings
☐	☒	For hierarchical and complex designs, identification of the appropriate level for tests and full reporting of outcomes
☐	☒	Estimates of effect sizes (e.g. Cohen's *d*, Pearson's *r*), indicating how they were calculated

Our web collection on statistics for biologists contains articles on many of the points above.

Software and code

Policy information about availability of computer code

Data collection	As described in the Methods section, event timestamps were collected using custom MATLAB R2016a code, and electrophysiological data were collected using Open Ephys
Data analysis	As described in the Methods section, data was analysed using custom MATLAB R2016a code which is available for review or reuse at https://github.com/EmmaRoscow/QlearningReplay and https://doi.org/10.5281/zenodo.17115788

For manuscripts utilizing custom algorithms or software that are central to the research but not yet described in published literature, software must be made available to editors and reviewers. We strongly encourage code deposition in a community repository (e.g. GitHub). See the Nature Portfolio guidelines for submitting code & software for further information.

Data

Policy information about availability of data

All manuscripts must include a data availability statement. This statement should provide the following information, where applicable:

- Accession codes, unique identifiers, or web links for publicly available datasets
- A description of any restrictions on data availability
- For clinical datasets or third party data, please ensure that the statement adheres to our policy

Behavioural data and preprocessed electrophysiological data are available at https://github.com/EmmaRoscow/QlearningReplay; data used in the manuscript figures and supplementary figures are included as a Source Data file

Research involving human participants, their data, or biological material

Policy information about studies with human participants or human data. See also policy information about sex, gender (identity/presentation), and sexual orientation and race, ethnicity and racism.

Reporting on sex and gender	No human data used
Reporting on race, ethnicity, or other socially relevant groupings	No human data used
Population characteristics	No human data used
Recruitment	No human data used
Ethics oversight	No human data used

Note that full information on the approval of the study protocol must also be provided in the manuscript.

Field-specific reporting

Please select the one below that is the best fit for your research. If you are not sure, read the appropriate sections before making your selection.

[x] Life sciences   [ ] Behavioural & social sciences   [ ] Ecological, evolutionary & environmental sciences

For a reference copy of the document with all sections, see nature.com/documents/nr-reporting-summary-flat.pdf

Life sciences study design

All studies must disclose on these points even when the disclosure is negative.

Sample size	The study consists of two cohorts, one of 6 rats to collect behavioural data, and one of 3 rats to collect behavioural and electrophysiological data. This is in line with similar studies in the literature, and the principle of 3 Rs for ethical use of animals, including use of computational modelling to extract maximal value from small sample sizes. No sample size calculation was performed
Data exclusions	No animals were excluded from the analyses. Some recording sessions were excluded from electrophysiological analysis if recordings were irretrievably corrupted. Poor-quality units (neurons) were also excluded from analysis, as detailed in the Methods
Replication	No direct replication attempted. This was a novel experiment that combined behavioural, electrophysiological and computational results to provide converging evidence rather than replicate. Methods are thoroughly described, and data and code used for analysis have been shared to enable independent replication by others
Randomization	Study involved only one experimental group for each cohort. Assignment of reward probabilities to spatial locations was pseudo-randomised between rats to avoid confounders, as detailed in the Results
Blinding	Study involved only one experimental group for each cohort, so blinding during data collection was not applicable. After data collection, the definition of experimental and control groups of neurons was central to the data analysis so could not be blinded

Reporting for specific materials, systems and methods

We require information from authors about some types of materials, experimental systems and methods used in many studies. Here, indicate whether each material, system or method listed is relevant to your study. If you are not sure if a list item applies to your research, read the appropriate section before selecting a response.

Materials & experimental systems

n/a	Involved in the study	
[x]	[ ]	Antibodies
[x]	[ ]	Eukaryotic cell lines
[x]	[ ]	Palaeontology and archaeology
[ ]	[x]	Animals and other organisms
[x]	[ ]	Clinical data
[x]	[ ]	Dual use research of concern
[x]	[ ]	Plants

Methods

n/a	Involved in the study	
[x]	[ ]	ChIP-seq
[x]	[ ]	Flow cytometry
[x]	[ ]	MRI-based neuroimaging

Animals and other research organisms

Policy information about studies involving animals; ARRIVE guidelines recommended for reporting animal research, and Sex and Gender in Research

Laboratory animals	Adult male Lister hooded rats, weighing 260g-430g, from Charles River Laboratories, UK
Wild animals	Study did not involve wild animals
Reporting on sex	Findings apply to male rats only. A single sex was selected to avoid an additional confounder of sex on results
Field-collected samples	Study did not involve samples collected from the field
Ethics oversight	All procedures were reviewed by the University of Bristol Animal Welfare and Ethical Review Board

Note that full information on the approval of the study protocol must also be provided in the manuscript.

Plants

Seed stocks	Study did not involve plants
Novel plant genotypes	Study did not involve plants
Authentication	Study did not involve plants
